# NADPH Oxidase-Derived Reactive Oxygen Species Are Involved in the HL-60 Cell Monocytic Differentiation Induced by Isoliquiritigenin

**DOI:** 10.3390/molecules171113424

**Published:** 2012-11-12

**Authors:** Hongmei Chen, Bo Zhang, Ying Yao, Na Chen, Xiaoyu Chen, Hui Tian, Zhenhua Wang, Qiusheng Zheng

**Affiliations:** 1 Key Laboratory of Xinjiang Endemic Phytomedicine Resources, Ministry of Education, School of Pharmacy, Shihezi University, Shihezi 832002, China; 2 Life Sciences School, Yantai University, Yantai 264005, China

**Keywords:** HL-60 cell, isoliquiritigenin, differentiation, NADPH oxidase, reactive oxygen species

## Abstract

The present study was undertaken to test the hypothesis that NADPH oxidase-derived reactive oxygen species (ROS) are involved in isoliquiritigenin (ISL)-induced monocytic differentiation in human acute promyelocytic leukemia HL-60 cells. Morphological changes, cell surface markers CD11b/CD14 and NBT-reducing ability were used to determine the differentiation of HL-60 cells, and 2,7-dichlorofluorescein (DCFH-DA) was used to detect the level of intracellular ROS. ISL-induced HL-60 cell differentiation was accompanied by an increase in the intracellular ROS levels. L-Buthionine-(*S*,*R*)-sulfoximine (BSO), *N*-acetyl-L-cysteine (NAC), superoxide dismutase (SOD) and 4-hydroxy-2,2,6,6-tetramethylpiperidinoxyl (Tempol) were used to interfere with ROS production. NADPH oxidase inhibitors, apocynin (APO) and diphenyleneiodonium (DPI) were used to study the role of NADPH oxidase in ISL-induced HL-60 cell differentiation. The ISL-induced HL-60 cell differentiation and intracellular ROS generation were enhanced by the oxidant BSO and inhibited by the antioxidants NAC, SOD, and tempol, and were also inhibited by the NADPH oxidase inhibitors APO and DPI. The protein and mRNA expression of the NADPH oxidase subunits gp91phox and p47phox were determined by Western blotting and RT-PCR, respectively. The levels of translation and transcription of the NADPH oxidase subunits gp91phox and p47phox increased markedly in a concentration-dependent manner. These findings suggest that NADPH oxidase plays a critical role in HL-60 cell differentiation induced by ISL and that NADPH oxidase-derived ROS is involved in the differentiation mechanism.

## Abbreviations

AO: acridine orange
APO: apocynin
BSO: L-buthionine-(*S*,*R*)-sulfoximine
DCF-DA: 2′,7′-dichlorofluorescein diacetate
DMSO: dimethyl sulfoxide
DPI: diphenyliodonium
ES: embryonic stem
HL-60: human promyelocytic leukemia cells
ISL: Isoliquiritigenin
MFI: mean fluorescence intensity
NAC: *N*-acetyl-L-cysteine
NADPH: nicotinamide adenine dinucleotide phosphate
NBT: Nitrobluetetrazolium
PBS: phosphate buffered saline
PCR: Polymerase Chain Reaction
ROS: reactive oxygen species
SMC: smooth muscle cell
SOD: superoxide dismutase
Tempol: 4-hydroxy-2,2,6,6-tetramethyl piperidinoxyl
TPA: 12-*O*-tetradecanoylphorbol 13-acetate

## 1. Introduction

Differentiation therapy, a novel and potentially less toxic form of cancer therapy, involves the use of agents that alone or in combination induce malignant reversion [1]. As a therapeutic strategy differentiation induction has had a powerful effect on hematopoietic malignancies, especially leukemia [2]. The human myelogenous leukemia cell line HL-60 originated from a patient with acute myeloblastic leukemia and is considered an excellent cell line for the *in vitro* study of cell differentiation because there are several distinct characteristics between untreated and differentiated cells [3,4,5]. Leukemia cells often exhibit features of immaturity, such as immature development and scattered, large, and irregular nuclei [6], lower nitrobluetetrazolium (NBT)-reducing ability [7], and weak expression of the cell surface markers CD11b and CD14 [8,9]. Upon treatment with differentiation inducers, such as D3, 1,25-(OH)_2_D_3_, and all-*trans* retinoic acid (RA), all or part of the malignant phenotypes mentioned above are reversed [10].

The family of nicotinamide adenine dinucleotide phosphate (NADPH) oxidases (NOX) has emerged as the major source of ROS induction in the membrane channel [11]. ROS may have a certain effect on the redifferentiation induced by drugs such as arsenic trioxide [12]. In fact, a relatively high level of ROS is necessary for cell differentiation and animal development [13,14]; however, the mechanisms involved remain unclear.

Isoliquiritigenin (ISL), a dietary flavonoid that is present in licorice, exhibits a variety of biological activities, including antioxidant, anti-inflammatory, chemo-preventive and antitumor properties. Previous studies indicated that ISL could protect dopaminergic neuronal cells and hippocampal neuronal cell through antioxidative effects [15,16]. ISL has also been reported to inhibit tumor cell proliferation and induce apoptosis [17]. In a previous study, we reported that the leukemia cells (HL-60) were induced to differentiate by isoliquiritigenin (ISL) in a concentration-dependent manner and that this differentiation was accompanied by changes in ROS levels [18]. Previous research showed that the NOX-derived ROS regulates angiotensin II-induced adventitial fibroblast differentiation into myofibroblasts [19]. Furthermore, Nox4-derived ROS are crucial for smooth muscle cell (SMC) differentiation from embryonic stem (ES) cells [20]. We assumed that NADPH oxidase-derived ROS is one potential mediator of the mature differentiation of HL-60 cells. However, the role of NADPH oxidase in ISL-induced HL-60 cell differentiation is not fully understood. In the current study, we attempt to explore the role of NADPH oxidase in ISL-induced HL-60 cell differentiation. 

## 2. Results and Discussion

### 2.1. ISL Induced Differentiation in HL-60 Cells

**Figure 1:**
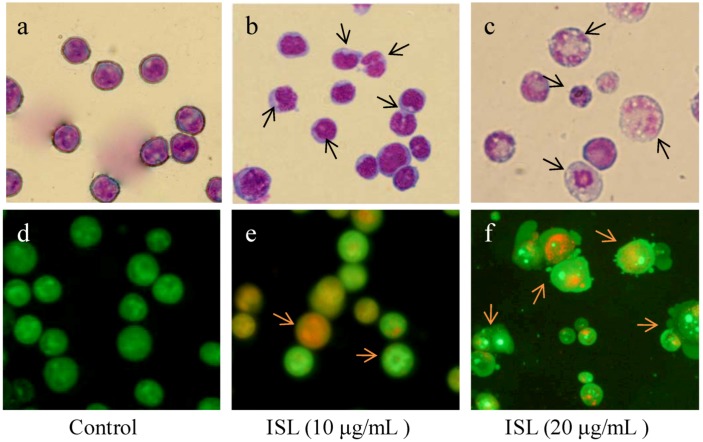
The effects of ISL on the morphology in HL-60 cells.HL-60 cells were treated with 10–20 μg/mL of ISL for 72 h. Cells were stained with Giemsa or AO and observed by microscopy (×40). The arrows indicate condensed nuclei. (**a**) Vehicle group; (**b**) ISL group (10 μg/mL);(**c**) ISL group (20 μg/mL) stained with Giemsa; (**d**) Vehicle group; (**e**) ISL group (10 μg/mL); (**f**) ISL group (20 μg/mL) stained with AO.

Untreated HL-60 cells displayed relatively large and round nuclei (Figure 1a,d). In contrast, 10 μg/mL ISL-treated cells were shrunken, with smaller and deformed nuclei (Figure 1b). When cells were treated with a higher concentration of ISL (20 μg/mL), condensed nuclei and apoptotic bodies were observed (Figure 1c,f). 

**Figure 2:**
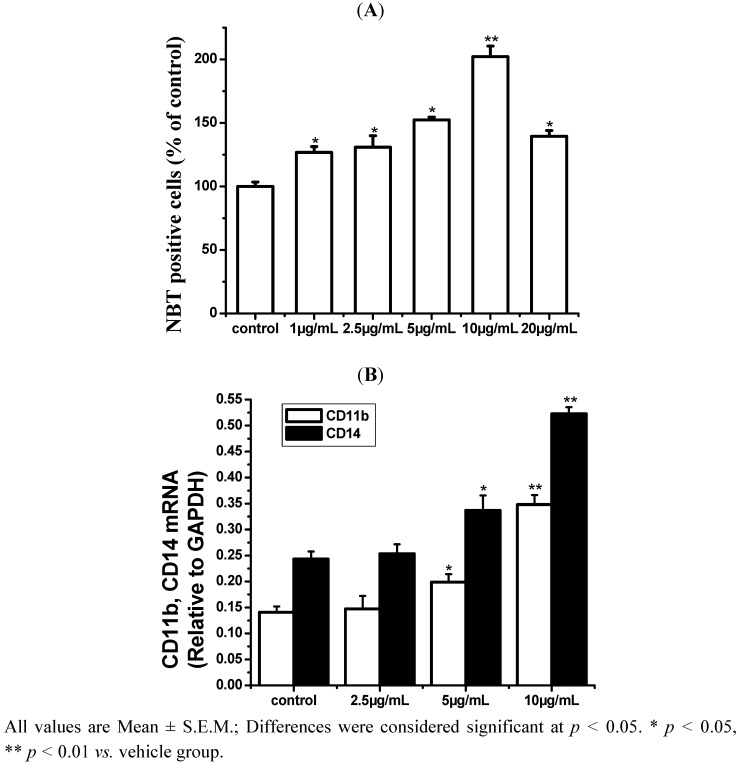
The effects of ISL on differentiation of HL-60 cells. (**A**) HL-60 cells were treated with 10–20 μg/mL of ISL for 72 h. The differentiation of HL-60 cells was determined by NBT absorbance at 590 nm/10^6^ cells. (**B**) Cells were treated with ISL (0, 2.5, 5, or 10 μg/mL) for 72 h, the levels of CD11b and CD14 mRNA expression. GAPDH was used as an internal control.

As shown in Figure 2, the number of NBT-positive cells significantly increased in an ISL concentration-dependent manner (2.5–10 μg/mL), peaked at 10 μg/mL, but declined after 72 h of treatment at 20 μg/mL. The levels of CD11b and CD14 mRNA expression were elevated markedly. These results suggested that the ISL-induced differentiation of HL-60 cells was optimal at a lower concentration of ISL; therefore, 2.5–10 μg/mL of ISL was chosen for further experiments.

### 2.2. ROS Is Closely Related to Cell Differentiation Induced by ISL

**Figure 3:**
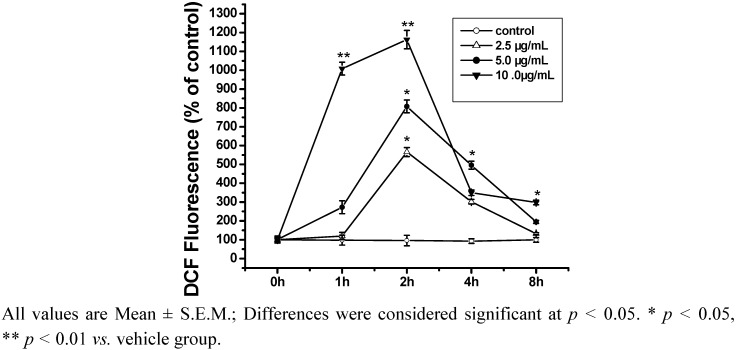
ROS production induced by ISL. HL-60 cells were treated with ISL 0–10 μg/mL for 1, 2, 4 or 8 h. Intracellular ROS levels were measured using DCF-DA.

Intracellular ROS production was determined by DCFH-DA probe. The levels of DCF fluorescence intensity in HL-60 cells were significantly increased during the first two hours of exposure to ISL (10 μg/mL) and then decreased significantly (Figure 3). The peak fluorescence was observed at 2 h. These results indicated that ISL induces the generation of ROS in a time-dependent manner.

**Figure 4:**
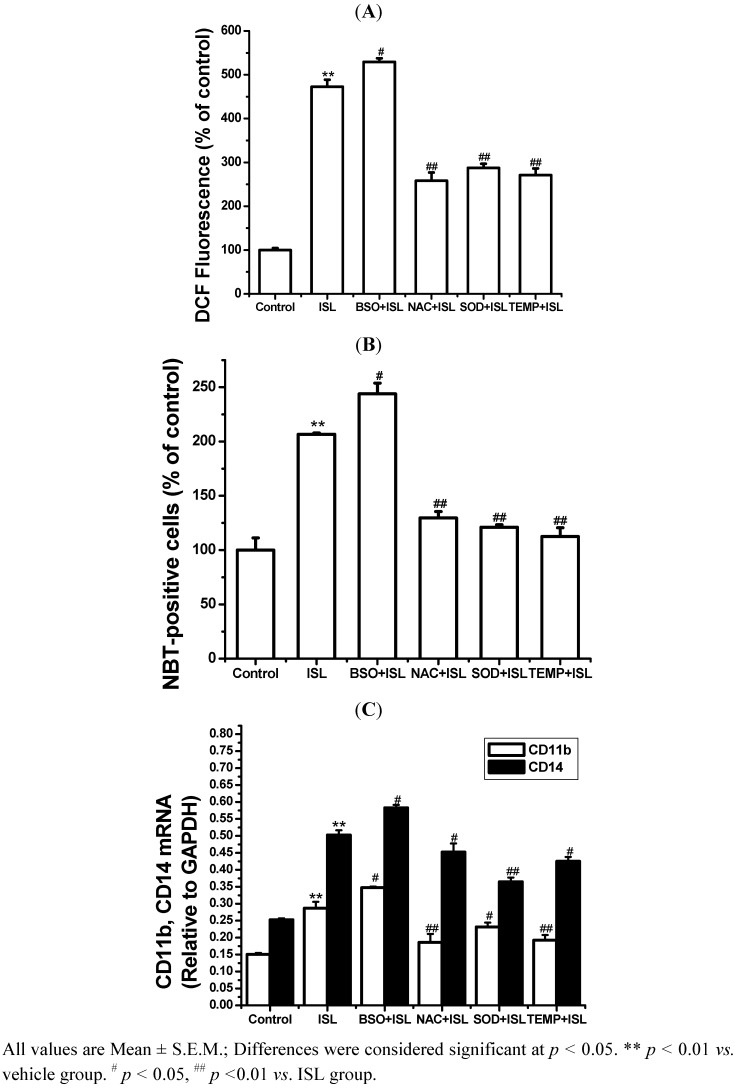
ROS production and NBT-positive cells induced by ISL. The cells were cultured with 8 μg/mL of ISL alone or in combination with NAC (500 μM), BSO (200 μM), SOD (200 μM), or tempol (200 μM) for 72 h, respectively. (**A**) Intracellular ROS levels were measured as described in the methods section. (**B**) Cell differentiation was determined and expressed as the percentage of NBT-positive cells relative to the number of viable cells (100%).(**C**) The levels of CD11b and CD14 mRNA expression. GAPDH was used as an internal control.

To verify that the increase of ROS level was involved in the differentiation, treatments with three antioxidants (NAC, SOD and Tempol) and one pro-oxidant (BSO) were performed. As shown in Figure 4, treatment by ISL combined with any one of the three antioxidants markedly decreased the ROS output and also inhibited the increase in NBT-positive cells and the levels of CD11b and CD14 mRNA expression, indicating the inhibition of cell differentiation; BSO had the opposite effect. These results indicated that the ROS level is closely related to the differentiation induced by ISL.

### 2.3. NADPH Oxidase Is Involved in Cell Differentiation Induced by ISL

**Figure 5:**
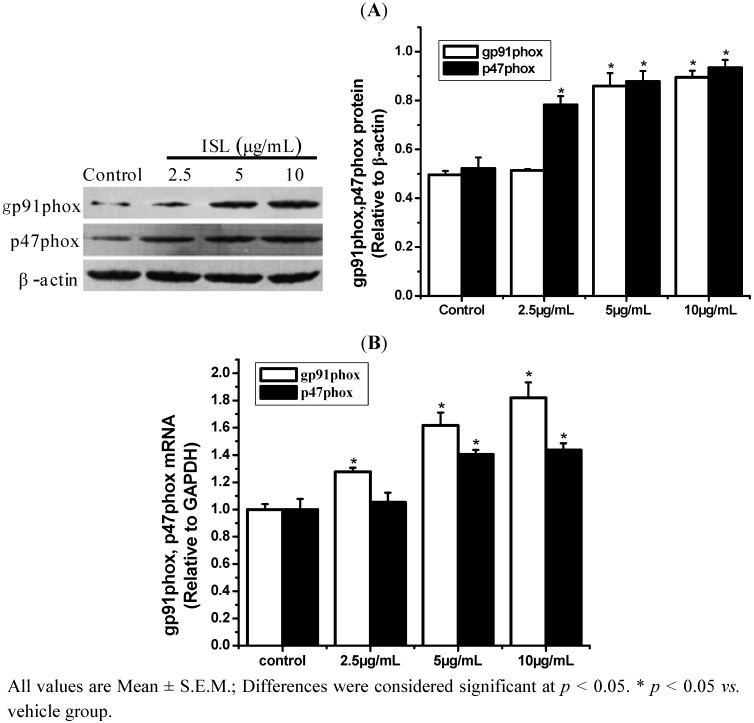
The effect of ISL on p47phox and gp91phox expression in HL-60 cells. (**A**) Cells were treated with ISL (0, 2.5, 5, or 10 μg/mL) for 72 h, and protein was extracted rapidly for Western blot analysis. β-Actin was used as the internal control. (**B**) The levels of p47phox and gp91phox mRNA expression in ISL-treated HL-60 cells. GAPDH was used as an internal control.

To determine whether the ROS production involves NADPH oxidase, the expression of the NADPH oxidase subunits gp91phox and p47phox at the translational (Figure 5A) and transcriptional (Figure 5B) levels are markedly increased in an ISL concentration-dependent manner (*p* < 0.05). The HL-60 cells were treated with the NADPH oxidase inhibitors APO or DPI alone or in combination with ISL.

**Figure 6:**
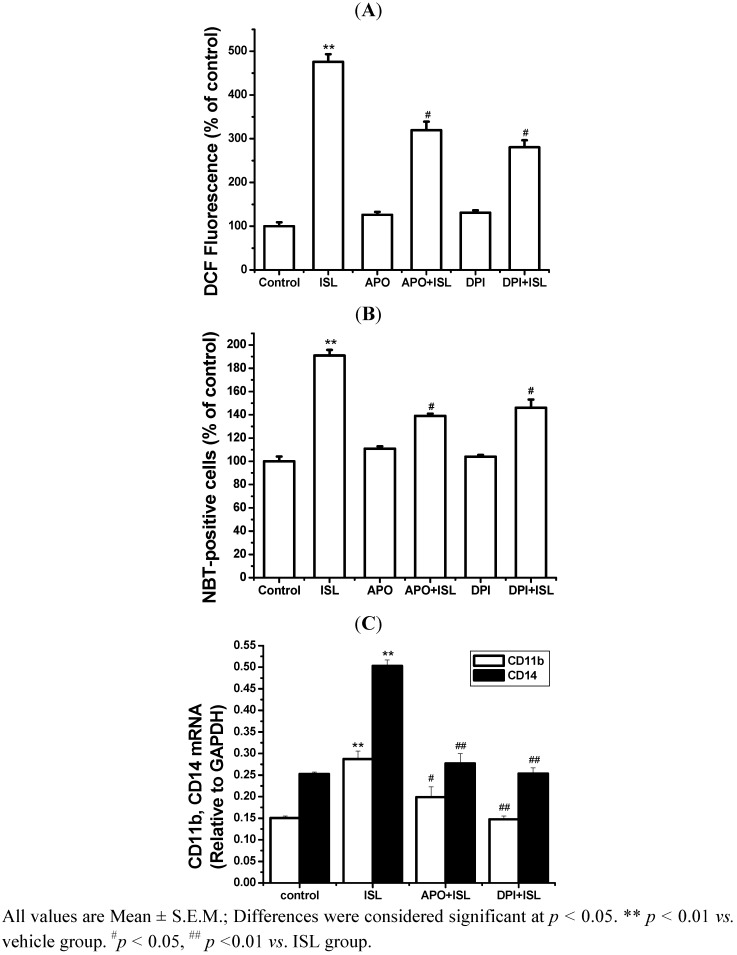
The effect of NADPH oxidase inhibition with APO and DPI on ROS production and differentiation in ISL-treated HL-60 cells. HL-60 cells cultured with 8 μg/mL of ISL were treated with or without 0.25 μM DPI or 0.5 mM APO for 72 h, respectively. (**A**) ROS was measured as described in the Experimental section. The fluorescence intensity is expressed as the percentage of vehicle control. (**B**) NBT-reducing activity was measured after 72 h. (**C**) The levels of CD11b and CD14 mRNA expression. GAPDH was used as an internal control.

APO (0.5 mM) or DPI (0.25 μM) markedly attenuated the production of ROS in ISL-treated cells, while treatment with APO or DPI alone had no effect on the production of ROS (Figure 6A). APO and DPI both significantly inhibited the increase in NBT-positive cells (Figure 6B) and the mRNA expression of cell surface markers CD11b and CD14 induced by ISL (Figure 6C). These findings indicated that the inhibition of NADPH oxidase inhibited cell differentiation. These results showed that ROS production is connected with NADPH oxidase in cell differentiation induced by ISL.

### 2.4. Discussion

In addition to cell morphology, the NBT-reducing ability and expression of the cell surface antigens CD11b/CD14 are considered specific markers of differentiation in HL-60 cells [18]. After treatment with ISL, the cells demonstrated morphological changes reflecting monocytic morphology, including shrunken and smaller cells and deformed nuclei. Moreover, the proportion of NBT-positive cells and the expression levels of CD11b and CD14 increased. This is convincing evidence that ISL induces monocytic differentiation in HL-60 cells. 

The intracellular ROS levels in HL-60 cells were significantly increased after exposure to ISL. As a potential pro-oxidant, the cytotoxicity of ISL to tumor cells may be due to its ability to form pro-oxidant phenoxyl radicals [21]. A few studies have focused on its chemical redox characteristics in noncellular conditions. However, ISL showed its dual roles on cellular redox status in a cellular system. In this study, the differention in the ISL-treated HL-60 cells was observed with the augmented intracellular ROS production at lower concentrations. The results were consistent with other reports, which showed that the proposed mode of action of hydroxyl chalcones was through the induction of the formation of pro-oxidant radicals, based on the studies in rat hepatocytes and human leukemia cells [22].

Further experiments showed that ISL combined with NAC, SOD or Tempol decreased ROS and inhibited cell differentiation. Meanwhile, ISL combined with BSO increased ROS and promoted cell differentiation. Our results not only indicated that ISL treatment induces the generation of ROS but also verified that the ROS level is closely related to HL-60 cell differentiation induced by ISL. It seems likely that many (and possibly all) anti-tumor agents cause oxidative stress, which also enhances their anti-cancer effects [23] because increased ROS formation contributes to the anti-tumor effects of chemotherapy. Of course, interfering with the ROS-antioxidant balance in cancers has risks. The administration of too many ROS generators could put normal cells at risk. For example, arsenic trioxide (As_2_O_3_) may act against acute promyelocytic leukemia by causing oxidative stress [24], but As_2_O_3_ can also be carcinogenic, and ROS are most likely involved in the mechanism of cancer development [25]. Direct damage to DNA is thought to be one key event, but it is insufficient to produce cancer, suggesting that the ability of ROS to suppress apoptosis and promote proliferation, invasiveness and metastasis (and possibly angiogenesis) are also important. Some reports have shown that redox homeostasis plays an important role in cell differentiation. An alteration in the redox homeostasis of the cells implies a change in ROS generation or metabolism. The pro-oxidant agent BSO promotes high levels of ROS generation by depleting reduced glutathione (GSH) [26]. NAC, a precursor of cysteine, elevates the GSH level, scavenges intracellular ROS, and protects against the initiation of apoptosis [27]. SOD, an enzymatic superoxide anion scavenger, and tempol, a non-enzymatic superoxide anion scavenger, decreases the output of superoxide anions to control the redox state of cells [2].

Cellular ROS production and NADPH oxidase are tightly correlated in cells [28], and NADPH oxidase is the major source of ROS induction in the membrane channel. NADPH oxidase consists of the catalytic subunit gp91phox, together with the regulatory subunits p22phox, p47phox, p40phox, p67phox and the small GTPase RAC. The p47phox regulatory subunit plays a critical role in activation of NADPH oxidase [29]. After ISL treatment, the levels of gp91phox and p47phox transcription and translation are markedly increased. Our findings indicated that when NADPH oxidase activity was inhibited, ROS production and cell differentiation were also inhibited. Malignant cells are often under self-inflicted (and/or host-inflicted) oxidative stress and that stress contributes to malignancy. One possible approach for tumor treatment would be to stress these cells further by providing agents that generate ROS. These should have limited effects on normal cells, provided that ROS levels do not rise too high, but might, when added to their already elevated generation of ROS, push malignant cells to a point at which the cell cannot cope with increasing oxidative damage. Indeed, it was observed that human leukemia cells are more sensitive to 2-methoxyoestradiol than are normal cells [30]. This agent appears to increase oxidative stress in cells, although its precise mechanism of action is not clear [31].

## 3. Experimental

### 3.1. Materials

ISL (purity 98%) was purchased from Jiangxi Herb Tiangong Technology Co., Ltd. (Yangling, Jiangxi Province, China), purity of isoliquiritigenin was analyzed according to our previous report [32]. Culture medium (RPMI 1640) and fetal bovine serum (FBS) were purchased from Gibco (Grand Island, NE, USA). Glutamine, 2,7-dichlorodihydrofluorescein diacetate (DCFH-DA), dimethyl sulfoxide (DMSO) and the anti-β-actin antibody were purchased from Sigma Chemical Co. (St. Louis, MO, USA). Penicillin and streptomycin were obtained from Shandong Sunrise Pharmaceutical Co., Ltd. (Zibo, Shandong Province, China). The anti-gp91phox antibody was obtained from Abcam Inc. (Cambridge, MA, USA); the anti-p47phox antibody was purchased from Santa Cruz Bio. (Santa Cruz, CA, USA). All other chemicals were of analytical grade and commercially available.

### 3.2. Cell Culture and Treatments

HL-60 human promyelocytic leukemia cells were purchased from the China Center for Type Culture Collection (Wuhan, China). The cells were kept in RPMI 1640 medium supplemented with 10% FBS, 50 U/mL penicillin, and 50 mg/mL streptomycin, as previously described [33]. HL-60 cells were grown in 5% CO_2_ and humidified in a 37 °C incubator. For each experiment, logarithmically growing HL-60 cells were used at a density 1 × 10^5^ cells/mL. Monocytic differentiation was induced by the addition of 2.5–10 μg/mL of ISL to the cell suspension [18].

### 3.3. Determination of Cell Differentiation

The treated cells were washed with cold phosphate-buffered saline (PBS) and fixed with methanol. After two washes with PBS, the cells were stained with Giemsa or acridine orange (AO). Cell differentiation was examined morphologically under a fluorescence microscope [34]. The degree of differentiation was assayed by the ability of the cells to reduce nitrobluetetrazolium (NBT) into insoluble blue-black formazan upon stimulation with phorbol-12-myristate-13-acetate (PMA) [35]. Each cell suspension (100 μL) was mixed with an equal volume of 2 mg/mL NBT dissolved in PBS containing 1 μg/mL PMA and incubated at 37 °C for 30 min. The reaction was stopped with the addition of 0.4 mL of cold 2 M HCl. The formazan product was obtained by centrifugation of the sample at 700 × *g* for 10 min. The supernatant was discarded, and the formazan was dissolved in 600 μL of DMSO. The percentage of NBT-positive cells with formazan deposits in the cytoplasm was determined by a fluorescence plate reader (Thermo Varioskan Flash 3001). The data are expressed as percentage of the control value.

### 3.4. Determination of Intracellular ROS

2,7-Dichlorodihydrofluorescein diacetate (DCFH-DA), a cell-permeable probe for intracellular ROS, is converted into DCF-DA by intracellular esterases and rapidly oxidized to the highly fluorescent DCF in the presence of intracellular hydrogen peroxide and peroxidases [36]. The treated cells were incubated for 15 min with 10 μM DCFH-DA and washed with PBS. DCF fluorescence was detected by a fluorescence plate reader (Thermo Varioskan Flash 3001). ROS levels were expressed as the mean fluorescence intensity (MFI) of DCF.

### 3.5. Western Blotting

After treatment, the HL-60 cells were washed twice with ice-cold PBS, lysed in RIPA buffer (lot 005, Sangon Co., Shanghai, China) at 4 °C for 1 h, and vortexed. Cell lysates were then cleared by centrifugation at 12,000 rpm for 10 min. The supernatant was collected, used for protein quantification and boiled for 5 min. Equal amounts (20 μg) of whole cell lysates were subjected to SDS-PAGE (10% polyacrylamide gel), transferred onto a nitrocellulose membrane and then probed with the appropriate primary and secondary antibodies before visualization using ECL Western blotting detection reagent [37] (Thermo) according to the manufacturer’s instructions.

### 3.6. RNA Extraction and Real-Time RT-PCR

Total RNA was extracted from HL-60 cells using an RNA extraction kit (Bio Basic Inc., Markham, ON, Canada) according to the supplier’s instructions. A 2 µg sample of total RNA was used for cDNA synthesis. Relative gene expression was quantified using the Mx3005P QPCR Detection System (Agilent Technologies Co. Ltd., Santa Clara, CA, USA) with SYBR-Green as a fluorescent dye, enabling the real-time detection of PCR products, according to the manufacturer’s protocol. The cDNA was submitted to real-time PCR using the primer pairs described below. The copy numbers of the cDNA targets were quantified using Ct values [38], and the mRNA expression levels of all samples were normalized to the level of the housekeeping gene GAPDH. Variations in the mRNA expression levels of different samples were evaluated as fold-induction compared with the untreated sample. The forward (F) and reverse (R) primers used to detect human transcripts were as follows (all 5′-3′): CD11b (GenBank Accession No.: NM_001145808.1) CD11bF: GGGAGTCCAACGCTAATG TC; CD11bR: GGGTCTGCTCGTAGTAATGG; CD14: (Accession No.: M86511.1) CD14F: CAAG CTCAGAGTGCTCGATC; CD14R: CCCGTCCAGTGTCAGGTTAT; gp91phox: (Accession No.: NM_000397.3) gp91phoxF: CCTAAGATAGCGGTTGATGG; gp91phoxR: GACTTGAGAATGGA TGCGAA; p47phox: (Accession No.: AF330627.1) p47phoxF: GTCAGATGAAAGCAAAGCGA; p47phoxR: CATAGTTGGGCTCAGGGTCT; GAPDH (Accession No.: NM_002046.3) GAPDHF: CCTCTGACTTCAACAGCGAC; GAPDHR: ACCAGGAAATGAGCTTGACA.

### 3.7. Statistical Analysis

The data obtained from different experiments are presented as the mean ± standard deviation of at least three independent experiments and were evaluated by ANOVA. The student’s *t*-test for multiple comparisons was used to identify differences among groups. Values were considered to be significantly different when *p* < 0.05.

## 4. Conclusions

These results confirmed the crucial role of NADPH oxidase in ISL-induced HL-60 cell differentiation and also suggested that ROS production is associated with the high expression of NADPH oxidase during the cell differentiation induced by ISL. One likely mechanism underlying these observations is that the ISL treatment caused mild oxidative stress in the HL-60 cells by up-regulating NADPH oxidase in a less ROS-sensitive manner, which leads to the induction of various secondary reactions. Further studies are necessary to investigate the role of redox homeostasis in cell differentiation induced ISL.
